# Assessment of electrical conductivity of polymer nanocomposites containing a deficient interphase around graphene nanosheet

**DOI:** 10.1038/s41598-024-59678-0

**Published:** 2024-04-16

**Authors:** Yasser Zare, Muhammad Tajammal Munir, Kyong Yop Rhee

**Affiliations:** 1 Biomaterials and Tissue Engineering Research Group, Department of Interdisciplinary Technologies, Breast Cancer Research Center, Motamed Cancer Institute, ACECR, Tehran, Iran; 2 College of Engineering and Technology, American University of the Middle East, 54200 Egaila, Kuwait; 3 Department of Mechanical Engineering (BK21 Four), College of Engineering, Kyung Hee University, Yongin, Republic of Korea

**Keywords:** Polymer graphene nanocomposites, Conductivity, Imperfect interface/interphase, Tunneling effect, Engineering, Materials science

## Abstract

In this study, a poor/imperfect interphase is assumed to express the effective interphase thickness, operative filler concentration, percolation onset and volume share of network in graphene–polymer systems. Additionally, a conventional model is advanced by the mentioned terms for conductivity of samples by the extent of conduction transference between graphene and polymer medium. The model predictions are linked to the experimented data. Likewise, the mentioned terms as well as the conductivity of nanocomposites are expressed at dissimilar ranges of various factors. The novel equations successfully predict the percolation onset and conductivity in the samples containing a poor/imperfect interphase. Thin and long nanosheets with high conduction transportation desirably govern the percolation onset and nanocomposite conductivity, but a bigger tunneling distance causes a lower conductivity.

## Introduction

Polymer nanocomposites containing graphene can be employed in dissimilar grounds such as energy devices, electromagnetic shielding, electronics, and light emitting diodes^1–9^. The conductivity in polymer nanocomposites is achieved above percolation onset in which the conductive networks of graphene are established^10^. The higher aspect ratio and larger surface area of graphene compared to CNT cause lower percolation onset promoting the electrical conductivity in nanocomposites^11^. Actually, strict conditions such as milling, sonication and chemical oxidization often break the CNTs to short rods. Additionally, waviness of CNTs in the nanocomposites reduces its aspect ratio and conductivity^12,13^. However, single-layer graphene has very high electrical conductivity and unlike CNTs, chirality is not a factor in its electrical conductivity^14^. These properties in addition to extremely high surface area demonstrate the great potential of graphene for improving the electrical conductivity of polymer composites.

Many variables such as filler amount, filler conduction, filler dimensions, dispersion of nanoparticles, tunneling effect and interfacial condition can manage the conductivity of polymer nanocomposites^15–17^. Some modes have been suggested for the conductivity of CNT-filled nanocomposites assuming the mentioned parameters^18–20^, but the modeling of conductivity for graphene systems is limited. The previous researchers have applied old equations for percolation and conductivity for graphene-filled examples^21–23^, nevertheless they improperly undertake the characters of nanoparticles in the conductivity. In fact, the previous models cannot take into account the novel parameters attributed to interphase and nanoparticles.

The interphase part is regularly built in nanocomposites, owing to the big interfacial space and robust polymer–filler interaction^24–26^. The significance of interphase on the rigidity of systems was debated in the earlier articles^27–29^. The interfacial/interphase properties can also impress the conductivity of samples (shortened as conductivity here), for the reason that they determine the extent of filler conduction transported to insulative medium. However, it was shown that many nanocomposites have a poor/imperfect interphase around nanoparticles^30–33^. A strong interface/interphase properly carries the significant filler conduction to medium, while low interfacial properties cannot do it. Furthermore, the interphase around particles can create the networked structures in polymer nanocomposites^34,35^. So, the interface/interphase part causes a main role in the percolation onset and nanocomposite conductivity (indicated as conductivity here). Few people have stated the key impacts of interphase on the toughness and percolation onset of composites^34,36^, but the effect of interphase on the conductivity was rarely investigated. Actually, the limited works for the conductivity in graphene examples have focused on the experimental data and conventional models, while the imperfect interphase mainly affects the nanocomposite conductivity and percolation onset.

The present article focuses on the imperfect/poor interphase to predict the conductivity for graphene-filled nanocomposites. The effective interphase thickness is expressed by the extent of conduction transference from conductive nanoparticles to the polymer medium. In addition, the operative filler fraction, percolation onset and net volume share are correlated to the extent of conduction transferring. Also, a model is suggested for nanocomposite conductivity by the extent of conduction transportation, tunneling properties and graphene size. The forecasts of the new model are assessed by experimented records. Moreover, the mentioned terms and nanocomposite conductivity are plotted against various parameters. Hopefully, our developed equations can replace the conventional ones to foretell the conductivity in graphene polymer products. All parameters and equations are simple, meaningful and reasonable guiding the researchers in this field.

## Modeling methods

The percolation onset in polymer graphite nanocomposites was formulated^37^ as:

1 $$ \phi_{p} = \frac{{27\pi D^{2} t}}{{4(\lambda + D)^{3} }} $$

“t” and “D” denote the thickness and diameter of nanosheets and “λ” displays the tunneling distance between neighboring nanosheets.

Nonetheless, D >> λ contracts this equation to:

2 $$ \phi_{p} = \frac{27\pi t}{{4D}} $$

The interphase and tunnels can decrease the percolation onset in nanocomposites, because the interphase around the graphene and the tunnels between nanoparticles reduce the space among nanosheets and facilitate the network production. Assuming these terms, the latter equation can be developed to:

3 $$ \phi_{p} = \frac{{27\pi t^{2} }}{{4tD + 2\left( {Dt_{i} + D\lambda } \right)}} $$

where “t_i_” is interphase thickness around nanosheets.

When the inverse aspect ratio (α = t/D) is assumed in Eq. (3), “$\phi_{p}$” is reformulated as:

4 $$ \phi_{p} = \frac{{\frac{{27\pi t^{2} }}{D}}}{{\frac{{4tD + 2\left( {Dt_{i} + D\lambda } \right)}}{D}}} = \frac{27\pi t\alpha }{{4t + 2t_{i} + 2\lambda }} $$

considering the dimensions of graphene, interphase and tunneling region in the percolation onset. Equation (4) was used to calculate the percolation onset for several graphene-filled samples in the previous articles^38–40^.

The interphase regions surrounding nanosheets also increase the effectiveness of nanoparticles in the samples. The interphase volume share^41^ is estimated by:

5 $$ \phi_{i} = \phi_{f} \left( {\frac{{2t_{i} }}{t}} \right) $$

where “$\phi_{f}$” is filler volume share.

The operative graphene volume share covers the contents of filler and interphase as:

6 $$ \phi_{eff} = \left( {1 + \frac{{2t_{i} }}{t}} \right)\phi_{f} $$

However, the imperfect interfacial properties between medium and nanoparticles restrict the transferring of conduction from filler to medium. This incidence deteriorates the conduction efficiency of nanoparticles and the nanocomposite conductivity.

In the case of imperfect/poor interfacial properties ($0 \le x \le D_{c}$), the whole diameter of nanosheet cannot reach the filler conduction (σ_f_), but complete interfacial adhesion ($D_{c} \le x \le D/2$) transfers the full conduction from nanoparticles to polymer medium. “D_c_” is expressed as the minimum diameter of nanosheets needed to transfer the complete conduction of nanofiller to polymer medium. In fact, 2D_c_ > D determines the poor interface, while 2D_c_ < D determines the perfect interface in the nanocomposites.

“D_c_” is suggested as:

7 $$ D_{c} = \frac{{\sigma_{{f_{{}} }} t}}{2\psi } = \frac{{\sigma_{{f_{{}} }} \alpha_{{}} D}}{2\psi } $$

where “ψ” signifies the interfacial conduction.

Assuming the interfacial properties, the effective diameter of nanosheets (D_eff_) is expressed by:

8 $$ \overline{\sigma } D = \sigma_{f} D_{eff} $$

As a result, the poor interfacial properties change the effective opposite aspect ratio (α_eff_) and the operative volume share ($\phi_{eff}$) of graphene in the products^31^ as:

9 $$ \alpha_{eff} = \alpha \left( {\frac{{8D_{c}^{2} }}{{D^{2} }} + 1} \right) $$

10 $$ \phi_{eff} = \phi_{f} \left[ {\frac{1}{2} + \left( {\frac{{1 - 4D_{c} }}{{2D^{{}} }}} \right)\frac{{\left( {1 - 4D_{c} } \right)}}{4D}} \right] $$

Additionally, the conduction transferring parameter among nanoparticles and medium can be defined by:

11 $$ Y = \frac{D}{{4D_{c} }} $$

Substituting of “Y” from the latter equation into Eqs. (9) and (10) presents the “α_eff_” and “$\phi_{eff}$” as:

12 $$ \alpha_{eff} = \alpha \left( {\frac{1}{{2Y^{2} }} + 1} \right) $$

13 $$ \phi_{eff} = \phi_{f} \left[ {\frac{1}{2} + \left( {1 - \frac{1}{2Y}} \right)\left( {1 - \frac{1}{4Y}} \right)} \right] $$

The calculations of Eq. (10) at average levels of all parameters show very slighter outputs than Eq. (6). Thus, Eqs. (10) and (13) can be modified to:

14 $$ \phi_{eff} = 5\phi_{f} \left[ {\frac{1}{2} + \left( {\frac{{1 - 4D_{c} }}{{2D^{{}} }}} \right)\frac{{(1 - 4D_{c} )}}{4D}} \right] = 5\phi_{f} \left[ {\frac{1}{2} + \left( {1 - \frac{1}{2Y}} \right)\left( {1 - \frac{1}{4Y}} \right)} \right] $$

Now, the effective interphase thickness can be correlated to “Y” by joining Eqs. (6) and (14) as:

15 $$ 1 + \frac{{2t_{i} }}{t} = 5\left[ {\frac{1}{2} + \left( {1 - \frac{1}{2Y}} \right)\left( {1 - \frac{1}{4Y}} \right)} \right] $$

Restructuring of this equation can express the effective interphase thickness by “t” and “Y” as:

16 $$ t_{i} = \frac{3t}{4} + \frac{5}{2}t\left( {1 - \frac{1}{2Y}} \right)\left( {1 - \frac{1}{4Y}} \right) $$

So, the effective interphase thickness depends on the graphene thickness and the conduction transportation between polymer and graphene.

When “α_eff_” and “t_i_” are replaced from Eqs. (12) and (16) into Eq. (4), “$\phi_{p}$” is suggested as:

17 $$ \phi_{p} = \frac{{27\pi t\alpha \left( {\frac{1}{{2Y^{2} }} + 1} \right)}}{{4t + 2\left[ {\frac{3t}{4} + \frac{5}{2}t\left( {1 - \frac{1}{2Y}} \right)\left( {1 - \frac{1}{4Y}} \right)} \right] + 2\lambda }} $$

considering the roles of conduction transferring parameter, graphene dimensions and tunneling distance in the percolation onset. The interphase factors such as “t_i_” and “Y” can be determined by Eqs. (16) and (17) when the experimentally measured percolation onset is available. The effective size range in which filler contact occurs is considered as t < 10 nm and D > 1 μm. In this condition, a low percolation onset encouraging the electrical conductivity in nanocomposites can be obtained.

Also, substituting of “t_i_” from Eq. (16) into Eq. (6) presents the operative filler fraction as:

18 $$ \phi_{eff} = \phi_{f} \left[ {1 + \frac{{\frac{3t}{2} + 5t\left( {1 - \frac{1}{2Y}} \right)\left( {1 - \frac{1}{4Y}} \right)}}{t}} \right] = \phi_{f} \left[ {1 + \frac{3}{2} + 5\left( {1 - \frac{1}{2Y}} \right)\left( {1 - \frac{1}{4Y}} \right)} \right] $$

The share of nanoparticles within the conductive networks after percolation onset^42^ can be given by:

19 $$ f = \frac{{\phi_{f}^{1/3} - \phi_{p}^{1/3} }}{{1 - \phi_{p}^{1/3} }} $$

The “f” term can be developed by “$\phi_{eff}$” (Eq. 18) and “$\phi_{p}$” (Eq. 17) as:

20 $$ f = \frac{{\phi_{eff}^{1/3} - \phi_{p}^{1/3} }}{{1 - \phi_{p}^{1/3} }} $$

Also, the volume share of net can be estimated by:

21 $$ \phi_{N}^{{}} = f\phi_{f}^{{}} $$

When “f” (Eq. 20) and “$\phi_{eff}$” (Eq. 18) are substituted in Eq. (21), “$\phi_{N}^{{}}$” is expressed by:

22 $$ \phi_{N}^{{}} = \frac{{\phi_{eff}^{1/3} - \phi_{p}^{1/3} }}{{1 - \phi_{p}^{1/3} }}\phi_{eff}^{{}} $$

stating that the concentration of networked nanosheets links to filler share and dimensions, tunneling distance and the extent of conduction transference.

In the next step, a conventional model is expanded for conductivity of graphene polymer examples by the cited terms.

Weber and Kamal^43^ suggested the longitudinal resistivity of composites as:

23 $$ \rho = \frac{{A_{f} \rho_{f} X}}{{\phi_{N} d_{c} l\cos^{2} \theta }} $$

where “A_f_” shows the cross-section area of fiber, “ρ_f_” is fiber resistivity, “d_c_” is diameter of tunneling area, “l” is length of fiber and “θ” denotes the angle between fiber and current direction.

“X” is defined as the quantity of contacts (m) as:

24 $$ X = \frac{1}{0.59 + 0.15m} $$

where the supreme “m” is 15.

The composite conductivity can be given by inverse “ρ” as:

25 $$ \sigma = \frac{{\phi_{N} d_{c} l\cos^{2} \theta }}{{A_{f} \rho_{f} X}} $$

which can be progressive for graphene-based nanocomposites by graphene characteristics, as mentioned.

Graphene cross-section area is calculated by:

26 $$ A_{f} = tD $$

Besides, the graphene conduction is stated by σ_f_ = 1/ρ_f_.

For 3D arbitrary dispersing of filler in the samples^44^, we get:

27 $$ \cos^{2} \theta = \frac{1}{3} $$

According to these equations, Eq. (25) can be promoted for graphene samples as:

28 $$ \sigma = \frac{{\phi_{N} d_{c} \sigma_{f} }}{3tX} $$

Nevertheless, this equation discounts the tunneling distance. Some researches have demonstrated that the conductivity adversely depends on the tunneling distance (λ)^9,40,45^. This assumption develops Eq. (28) to:

29 $$ \sigma = \frac{{\phi_{N} d_{c} \sigma_{f} }}{{3tX\left( {\frac{\lambda }{z}} \right)^{3} }} $$

where “z” as tunneling factor is 0.1 nm.

Switching of “$\phi_{N}^{{}}$” from Eq. (22) into Eq. (29) establishes an advanced model for conductivity as:

30 $$ \sigma = \frac{{\frac{{\phi_{eff}^{1/3} - \phi_{p}^{1/3} }}{{1 - \phi_{p}^{1/3} }}\phi_{eff} d_{c} \sigma_{f} }}{{3tX\left( {\frac{\lambda }{z}} \right)^{3} }} $$

which shows the significances of various parameters for nanoparticles, poor/imperfect interphase and tunneling region on the conductivity. All parameters included in Eq. (30) are meaningful and determinate facilitating the prediction of nanocomposite conductivity by poor/imperfect interphase. The proposed equation considers the various dimensions of graphene nanosheets in the samples. The proposed equations are valid when the thickness of graphene is less than 10 nm and graphene diameter is more than 1 μm. This range of graphene dimensions provides the low percolation onset and good electrical conductivity in the nanocomposites.

## Results and discussion

### Evaluation of model by experimental data

The predictions of the suggested equations are matched to the measured results of some examples for evaluation and estimation of parameters. Table 1 expresses four examples and their characteristics from original references. Besides, the percolation onset was obtained by the conductivity measurements in the original references. By application of Eq. (17) to the experimented facts, “λ” and “Y” are attained estimating the effective interphase thickness (t_i_) for the reported samples by Eq. (16). All calculations are shown in Table 1.

**Table 1:** Investigated samples, their characteristics and parameters calculations using advanced equations.

Refs	Samples	t (nm)	D (μm)	$\phi_{p}$	λ (nm)	Y	t_i_ (nm)	d_c_ (nm)	m
^46^	PS/graphene	1	2	0.0010	12	8	3	150	85
^47^	SAN/graphene	1	2	0.0017	7	5	2.9	10	20
^48^	PS/graphene	1	4	0.0005	13	15	3.1	450	925
^47^	ABS/graphene	1	4	0.0013	3	3	2.7	1	2
^49^	PVA/graphene	2	2	0.0035	13	22	6.3	22	15
^50^	PET/graphene	2	2	0.0050	7	10	6.1	50	39

**Figure 1:**
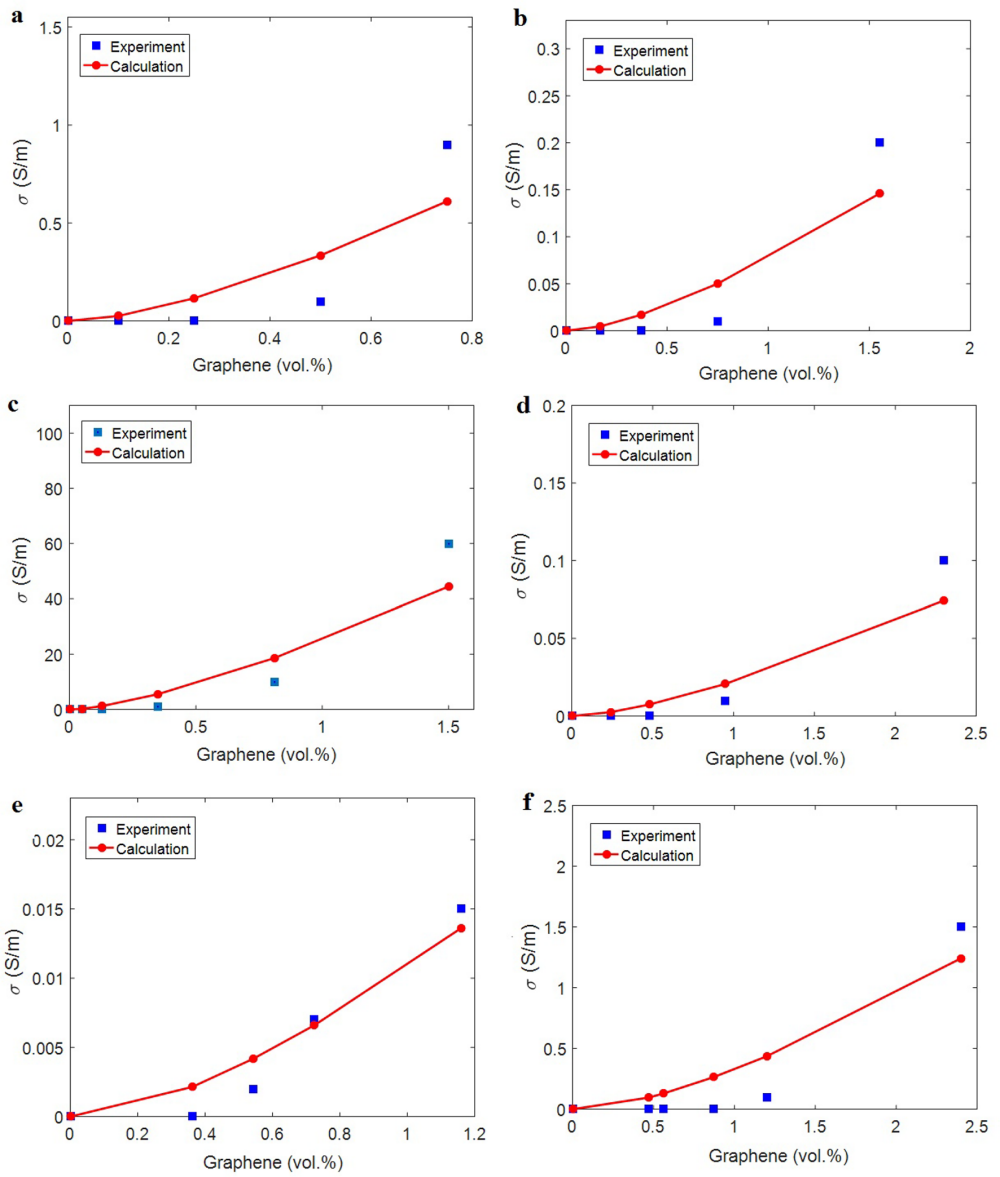
The experimented and predicted conductivity by the new model for (**a**) PS^46^, (**b**) SAN^47^, (**c**) PS^48^, (**d**) ABS^47^, (**e**) PVA^49^ and (**f**) PET^50^ graphene products.

The largest tunneling distance, the highest level of conduction transportation between nanoparticles and polymer medium and the thickest interphase are shown in PVA/graphene sample, while the least levels of these parameters are observed in ABS/graphene nanocomposite. The calculated ranges of parameters show the key effects of tunneling region, conduction transference level and interphase on the percolation onset. However, the conventional equations only considered the characters of filler size in the percolation onset, which is not sufficient for nanocomposites, because many parameters such as “λ”, “Y” and “t_i_” govern the percolation onset. The calculations of parameters are used in Eq. (30) for predicting of conductivity. Figure 1 portrays the experimented facts and the model predictions for the examples. The outputs are matched to the experimented values, which approve the predictability of the model. Therefore, the model can positively predict the conductivity. In fact, the new model is applicable for the conductivity assuming tunneling region between sheets, conduction transportation from nanoparticles to medium and poor/imperfect interphase section. The values of “d_c_” and “m” are also reported in Table 1. The highest levels of “d_c_” and “m” are reported for PS/graphene sample (No. 3), while the minimum levels of these parameters are shown in ABS/graphene nanocomposite. All parameters are meaningful and reasonable validating the proposed model.

### Parametric examinations

In this section, parametric investigations are carried out to confirm the proposed equations.

**Figure 2:**
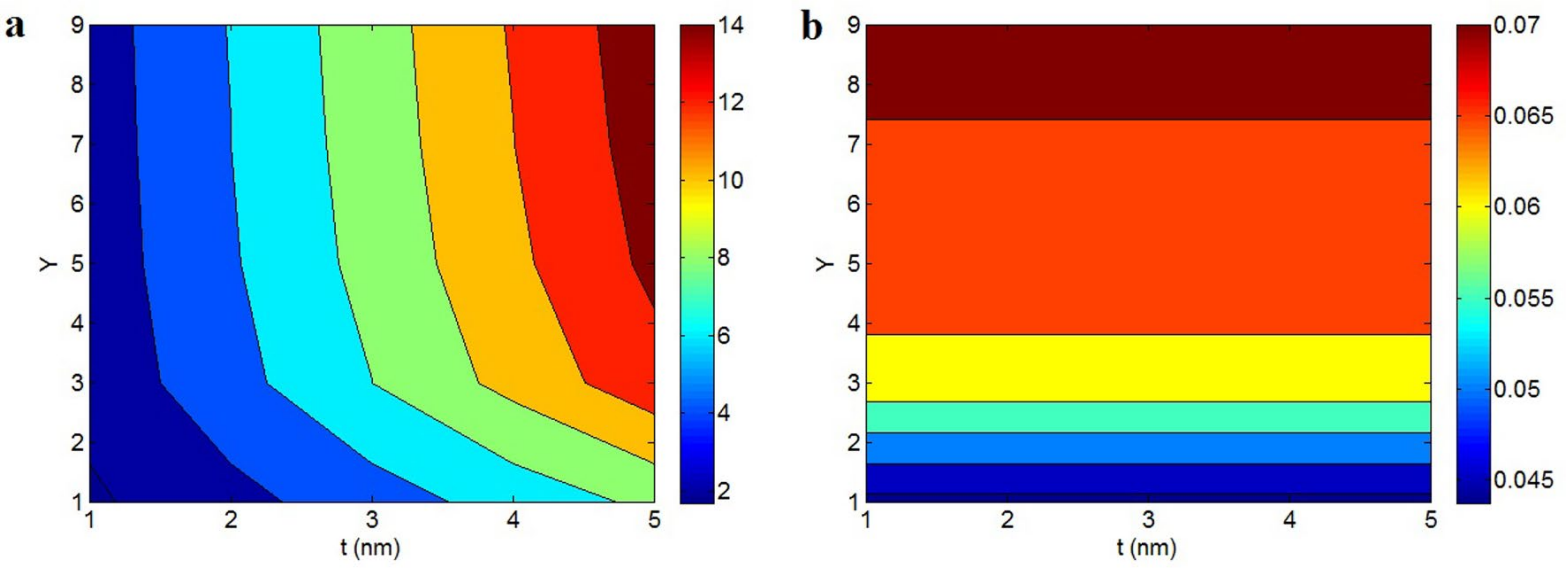
Dependencies of (**a**) effective interphase thickness (Eq. 16) and (**b**) “$\phi_{eff}$” (Eq. 18) on “t” and “Y”.

Figure 2a displays the roles of “t” and “Y” in the effective interphase depth by contour plot via Eq. (16). The thickest interphase as 14 nm is shown at t = 5 nm and Y > 4.2, whereas the thinnest interphase as 2 nm is predicted by t = 1 nm and Y = 1. Therefore, thick nanosheets and high conduction transportation between nanoparticles and medium achieve a desirable effective interphase thickness. However, the effective interphase thickness decreases by thin nanosheets and low “Y”.

The effective interphase thickness shows a direct link to the thickness of graphene nanosheets according to Eq. (16). As a result, thick nanosheets produce a thick interphase in graphene nanocomposites. Moreover, it is clear that the conduction transference rightly links to the interfacial/interphase aspects. So, a big conduction of transportation shows the high interfacial/interphase features, which produce a thick interphase around nanoparticles. In other words, a thicker interphase demonstrates the higher level of interfacial/interphase properties in nanocomposites results in better conduction transportation. Accordingly, Eq. (16) correctly expresses the correlation of effective interphase thickness to “t” and “Y”.

Figure 2b exemplifies the impacts of “t” and “Y” on the operative filler share ($\phi_{eff}$) at $\phi_{f}$ = 0.01 based on Eq. (18). “t” cannot change the operative filler share, but “Y” directly controls the “$\phi_{eff}$”. The highest and the least “$\phi_{eff}$” as 0.07 and 0.044 are calculated at Y > 7.5 and Y = 1, demonstrating that the conduction transference importantly manages the effectiveness of nanoparticles in the products.

According to Eq. (18), it is obvious that the effective filler concentration does not depend on the thickness of nanosheets. However, a high transportation of conduction from nanoparticles to polymer medium results in proper assignment of filler conduction to insulative medium promoting the conductivity. Instead, a low “Y” expresses the low transportation of conduction to polymer medium, which deteriorates the conduction efficiency of nanoparticles. Consequently, the effectiveness of nanoparticles mainly links to the “Y”, as suggested by Eq. (18).

**Figure 3:**
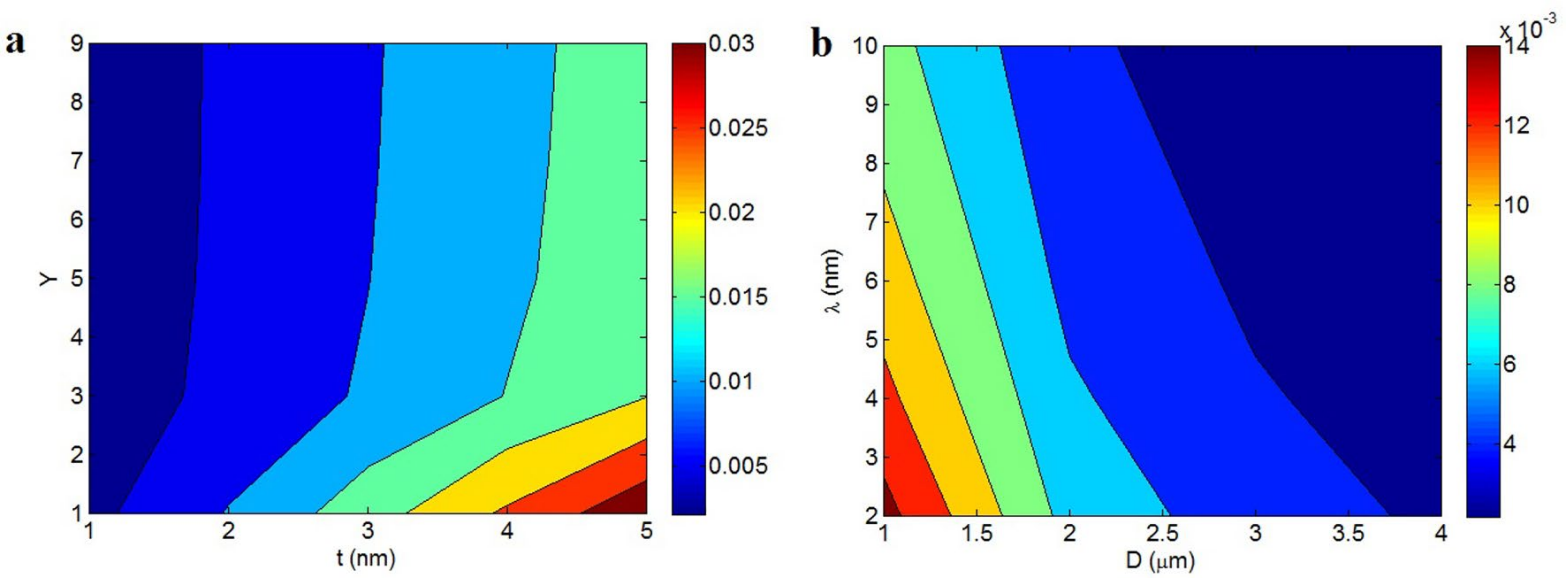
Percolation onset as a function of (**a**) “t” and “Y” and (**b**) “D” and “λ” by Eq. (17).

Figure 3a shows the variation of percolation onset at unalike ranks of “t” and “Y” at D = 2 μm and λ = 5 nm (Eq. 17). The maximum “$\phi_{p}$” as 0.03 is witnessed at t = 5 nm and Y = 1, although the smallest $\phi_{p}$ = 0.001 is shown by t < 1.7 nm and Y > 3. These results indicate that thin nanosheets and high conduction transportation cause a desirable percolation level in nanocomposites, while thick nanosheets and weak conduction transference increase it.

Thin nanosheets yield a big quantity of particles in a unit volume, which increases the possibility of networking. Accordingly, thin nanosheets cause a small percolation onset in the system. However, thick nanosheets weaken the contact number among sheets and induce a high percolation onset, because the percolating of filler needs the contacts between nanoparticles. Additionally, high conduction transference displays the desirable levels for interfacial/interphase properties in nanocomposites. Since the interfacial/interphase properties directly affect the percolation onset, a higher level of “Y” produces a lower percolation level in nanocomposites. In fact, the interphase regions can produce the percolated structures in nanocomposites and thus, large interphase regions due to tough interfacial interactions positively handle the percolation onset.

The powers of “D” and “λ” on the percolation onset are also revealed in Fig. 3b at t = 2 nm and Y = 5. The smallest percolation level as about 0.002 is observed at D > 2.5 μm and λ > 8 nm, but a high percolation onset as 0.014 is realized by D = 1 μm and λ = 2 nm. So, big nanosheets and large tunnels can desirably control the percolation onset in composites. The big nanosheets cause numerous contacts in nanocomposites, because they are separated by small distances. As a result, their networking is easier than that of short nanosheets producing a low percolation onset. Besides, a large tunneling distance between nanosheets can reduce the percolation onset, since the separated nanosheets by tunneling distance can establish the conductive networks. However, in the case of short tunneling distance, only few adjacent nanosheets can take part in the networks shifting the percolation onset to high filler concentrations. According to these reasons, big nanosheets and large tunneling distance logically present a low percolation onset.

**Figure 4:**
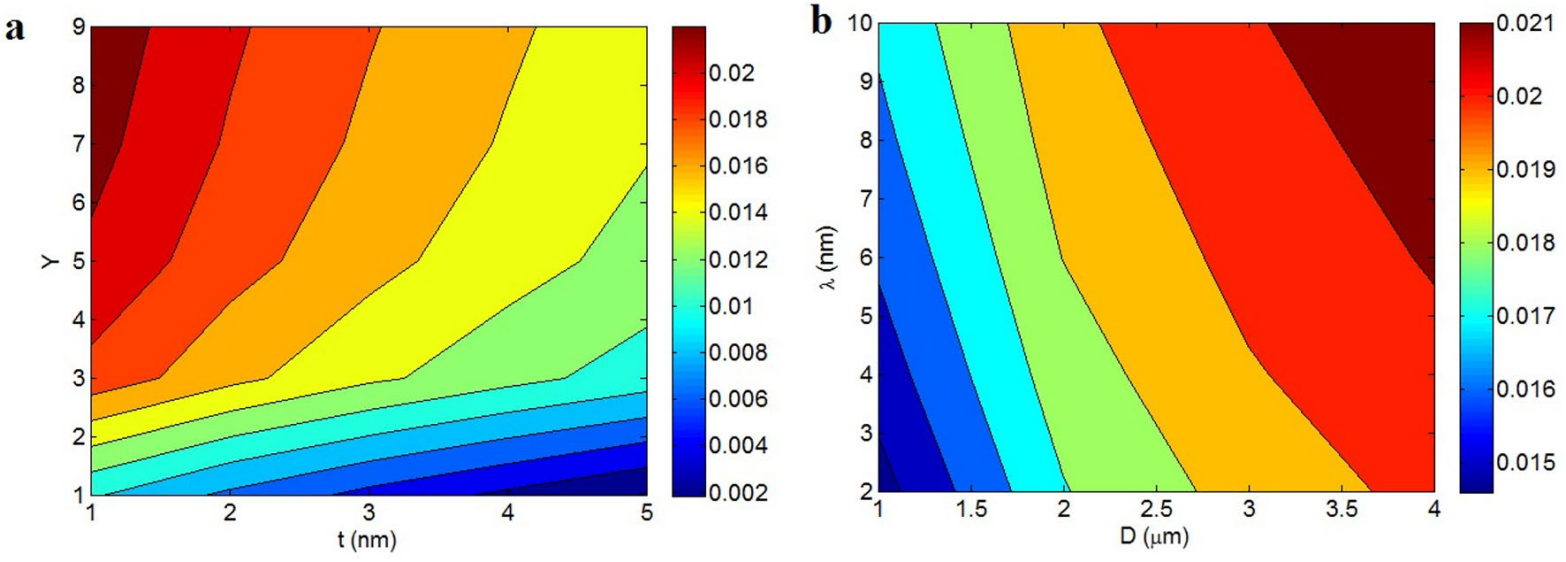
Linking of “$\phi_{N}$” (Eq. 22) to (**a**) “t” and “Y” and (**b**) “D” and “λ”.

The volume portion of percolated graphene ($\phi_{N}$) at various ranges of “t” and “Y” and $\phi_{f}$ = 0.01, D = 2 μm and λ = 5 nm is also illustrated in Fig. 4a based on Eq. (22). The maximum level of “$\phi_{N}$” as 0.022 is obtained by t = 1 nm and Y > 6, but “$\phi_{N}$” mainly decreases to 0.002 at t > 4 nm and Y = 1. Therefore, thin nanosheets and high conduction transportation can grow the share of networked nanosheets, while thick nanosheets and poor transferring of conduction reduce it.

Thin nanosheets decrease the percolation onset in nanocomposites. In fact, they show a high potential for percolating, because they increase the level of inter-contacts. So, it is sensible to get a big “$\phi_{N}$” by thin nanosheets. In addition, a high conduction transference declines the percolation onset and increases the operative filler share, because it reveals the significant levels of interfacial/interphase parameters in nanocomposites. Since a high share of nets is obtained by poor percolation onset and high operative filler share, it is correct to obtain a high “$\phi_{N}$” by great “Y”. In fact, the high transportation of conduction obtained by strong interfacial interactions facilitates the networking of nanosheets in nanocomposites.

The correspondence of “$\phi_{N}$” to “D” and “λ” (Eq. 22) at $\phi_{f}$ = 0.01, t = 2 nm and Y = 5 is also plotted in Fig. 4b. The highest $\phi_{N}$ = 0.021 is found by D > 3.5 μm and λ > 8 nm, nevertheless the minimum “$\phi_{N}$” as 0.0147 is observed at D = 1 μm and λ = 2 nm. Hence, “D” and “λ” directly control the network volume share in nanocomposites.

Big nanosheets are easily percolated in nanocomposites, because a small distance is existed between them. As a result, a small number of big nanosheets can construct the nets, which positively affect the volume share of networked nanosheets. In other words, larger nanosheets are easily networked compared to shorter ones resulting in the large networks in nanocomposites. Also, larger tunneling distance produces a smaller percolation level. In fact, a big sum of sheets can participate in the nets by large tunneling distance, which grows the net share. Accordingly, the high ranges of both “D” and “λ” desirably affect the volume share of nanosheets, as articulated by the advanced equation.

**Figure 5:**
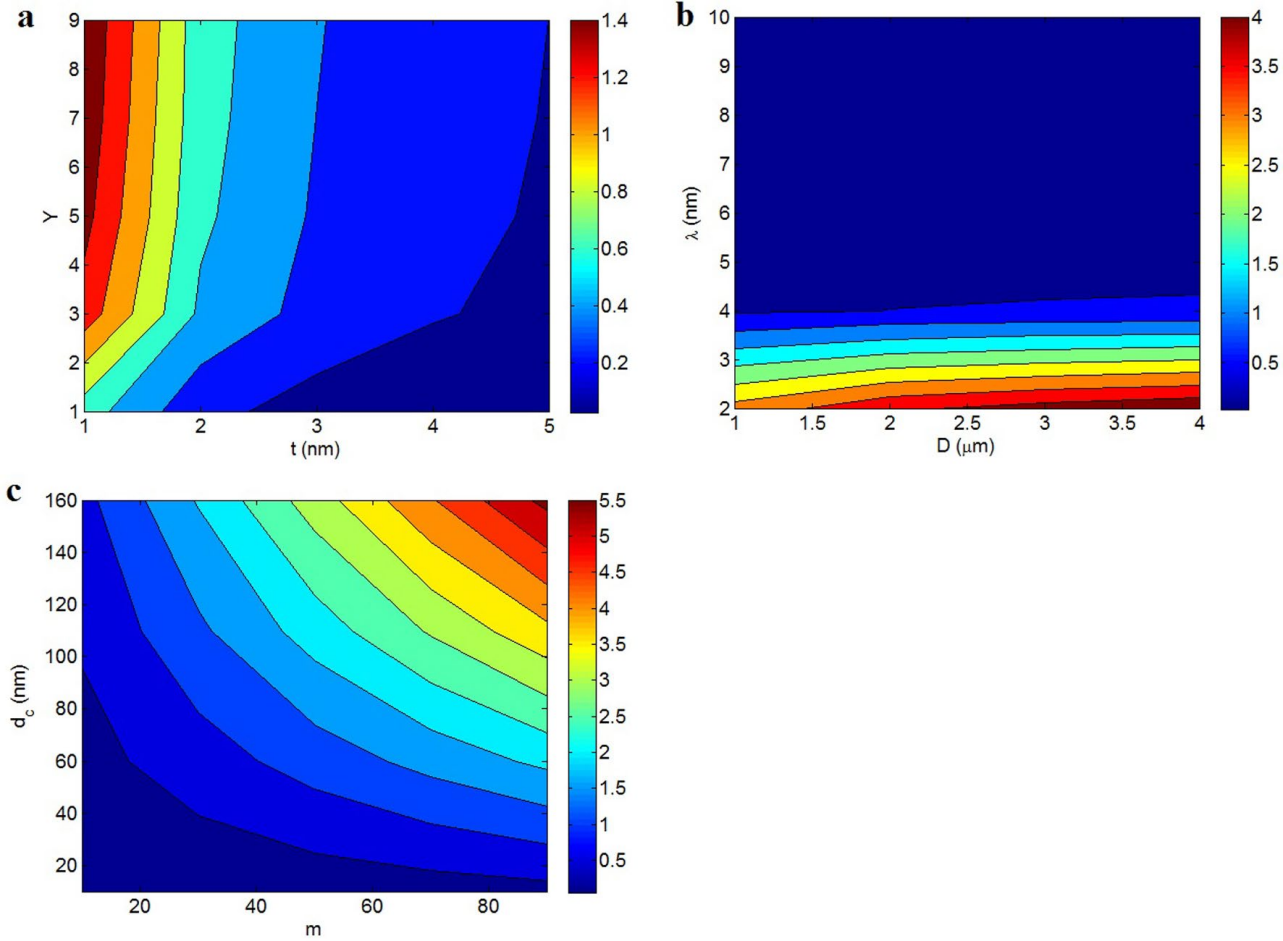
Calculations of the suggested model (Eq. 30) at various extents of (**a**) “t” and “Y”, (**b**) “D” and “λ” and (**c**) “m” and “d_c_”.

Figure 5 reveals the influences of numerous parameters on the conductivity by Eq. (30). Figure 5a shows the conductivity by “t” and “Y” at σ_f_ = 10^5^ S/m, $\phi_{f}$ = 0.01, D = 2 μm, λ = 5 nm, d_c_ = 50 nm and m = 30. The supreme conductivity of 1.4 S/m is shown at t = 1 nm and Y > 4, nevertheless an insulative material is witnessed at t > 4 nm and Y < 3. Accordingly, the conductivity mainly links to the thickness of nanosheets and the extent of conduction transportation between polymer medium and nanoparticles. As observed, thin nanosheets and high “Y” obtain a high conductivity.

Thin nanosheets move the percolation onset to poor filler amounts. Also, a high share of thin nanosheets can partake to the nets. As known, the size of networked nanosheets considerably manipulates the conductivity, since the nets carry the charges. Consequently, thin nanosheets increasing the size of nets improve the conductivity. Furthermore, a high conduction transfer can significantly allocate the graphene conduction to insulative polymer medium, which raises the conductivity. Conversely, a little “Y” due to the weak interfacial/interphase declines the efficiency of nanoparticles in the conductivity of samples, because the filler conduction cannot be assigned to polymer medium. So, the novel model truly states the correlation of conductivity to “t” and “Y”.

The predictions of nanocomposite conductivity at numerous ranges of “D” and “λ” are depicted in Fig. 5b. The top conductivity of 4 S/m is realized by D > 2.5 μm and λ = 2 nm, but λ > 4 nm seriously diminishes the conductivity. Therefore, large nanosheets and short tunnels get a high conductivity, while large tunnels cannot improve the conductivity.

Large nanosheets positively regulate the percolation onset and the volume share of nets based on the earlier discussion. Therefore, it is clear to take a better conductivity by larger nanosheets, because the big nanosheets enhance the dimensions of nets. Also, a large tunneling distance can positively handle the percolation onset and the share of nets, as mentioned in the previous illustrations. However, a large tunnel weakens the transferring of charges, since distant nanosheets cannot transport the charges^51^. Accordingly, a big tunnel ineffectively transfers the charges in the nanocomposites that deteriorate the conductivity. As well, a large tunnel can seriously decrease the conductivity, because the electrical conductivity links to the transportation of electrons through nets. Based on these reasons, the presented model fittingly displays the powers of “D” and “λ” on the conductivity.

Figure 5c represents the conductivity at various ranges of “m” and “d_c_”. The highest conductivity of 5.5 S/m is got by m = 90 and d_c_ = 160 nm, nevertheless the conductivity reduces to 0 at m < 30 and d_c_ < 40 nm. Also, very low levels of “d_c_” produce an insulative nanocomposite. Consequently, a large number of contacts and big contact zone between adjacent nanosheets obtain an appropriate conductivity, while few contacts among nanoparticles and poor contact area cannot raise the conductivity.

The high quantity of contacts increases the networking possibility in the system. In fact, the nanosheets can construct the filler networks when they have strong contacts. So, the contacts between nanosheets confidently affect the net size and the conductivity. Moreover, the contact area between neighboring nanosheets is necessary to establish the tunneling region. A huge contact area mainly declines the contact resistance between nanosheets^52^. Thus, a big contact area intensifies the transferring of electrons thorough the adjacent nanosheets improving the conductivity. On the other hand, short contact area between nanosheets increases the contact resistance, which reduces the electron current via contact regions. Thus, the contact zone directly manages the conductivity, because it manipulates the electron moving through tunnels. This explanation presents the proper stimuli of “m” and “d_c_” on the conductivity confirming the predictions of suggested model.

## Conclusions

In the case of a poor/imperfect interphase, the extent of conduction transference from graphene to polymer matrix was applied to express the effective interphase thickness, operative filler share, percolation onset and volume share of nets. Additionally, a conventional model was developed for nanocomposite conductivity by the extent of conduction transportation, tunneling properties and graphene dimensions. The estimations of new model were evaluated by the measured data. Also, the mentioned terms as well as the conductivity were plotted at different series of various parameters. Thin and large nanosheets along with high conduction transportation advantageously govern the percolation onset, the portion of network and the nanocomposite conductivity. However, although large tunnels decline the percolation onset and improve the net fraction, it weakens the electron transference between adjacent nanosheets and deteriorates the conductivity. Furthermore, a large number of contacts and big contact area between neighboring nanosheets produce a high conductivity. The impresses of all parameters on the mentioned terms and nanocomposite conductivity were evaluated and discussed approving the predictability of the suggested equations. Accordingly, the developed equations can appropriately calculate the percolation onset and conductivity for graphene-containing samples.

## Data Availability

All data generated or analyzed during this study are included in this published article.

## References

[1] Basha, I. K., Abd El-Monaem, E. M., Khalifa, R. E., Omer, A. M. & Eltaweil, A. S. Sulfonated graphene oxide impregnated cellulose acetate floated beads for adsorption of methylene blue dye: Optimization using response surface methodology. *Sci. Rep.* **12**(1), 9339 (2022). 35660768 10.1038/s41598-022-13105-4 PMC9167308

[2] Farouq, R. Functionalized graphene/polystyrene composite, green synthesis and characterization. *Sci. Rep.* **12**(1), 21757 (2022). 36526669 10.1038/s41598-022-26270-3 PMC9756699

[3] Mantecón-Oria, M. *et al.* Influence of the properties of different graphene-based nanomaterials dispersed in polycaprolactone membranes on astrocytic differentiation. *Sci. Rep.* **12**(1), 13408 (2022). 35927565 10.1038/s41598-022-17697-9 PMC9352708

[4] Md Said, N. H. *et al.* Review of the past and recent developments in functionalization of graphene derivatives for reinforcement of polypropylene nanocomposites. *Polym. Compos.* **42**(3), 1075–1108 (2021).

[5] Trivedi, D. N. & Rachchh, N. V. Graphene and its application in thermoplastic polymers as nano-filler—A review. *Polymer* **240**, 124486 (2021).

[6] Najafi rad, Z., Farzad, F. & Razavi, L. Surface functionalization of graphene nanosheet with poly (l-histidine) and its application in drug delivery: Covalent vs non-covalent approaches. *Sci. Rep.* **12**(1), 19046 (2022). 36351935 10.1038/s41598-022-21619-0 PMC9646737

[7] Veiskarami, A., Sardari, D., Malekie, S., Mofrad, F. B. & Kashian, S. Evaluation of dosimetric characteristics of a ternary nanocomposite based on high density polyethylene/bismuth oxide/graphene oxide for gamma-rays. *Sci. Rep.* **12**(1), 18798 (2022). 36335163 10.1038/s41598-022-23605-y PMC9637186

[8] Velo, M. M. A. C. *et al.* Enhancing the mechanical properties and providing bioactive potential for graphene oxide/montmorillonite hybrid dental resin composites. *Sci. Rep.* **12**(1), 10259 (2022). 35715426 10.1038/s41598-022-13766-1 PMC9205868

[9] Zare, Y. & Rhee, K. Y. Effect of contact resistance on the electrical conductivity of polymer graphene nanocomposites to optimize the biosensors detecting breast cancer cells. *Sci. Rep.* **12**(1), 1–10 (2022). 35354877 10.1038/s41598-022-09398-0 PMC8967928

[10] Martin-Gallego, M., Bernal, M., Hernandez, M., Verdejo, R. & Lopez-Manchado, M. Comparison of filler percolation and mechanical properties in graphene and carbon nanotubes filled epoxy nanocomposites. *Eur. Polym. J.* **49**(6), 1347–1353 (2013).

[11] Xie, S., Liu, Y. & Li, J. Comparison of the effective conductivity between composites reinforced by graphene nanosheets and carbon nanotubes. *Appl. Phys. Lett.* **92**(24), 243121 (2008).

[12] Zare, Y. & Rhee, K. Y. Significances of interphase conductivity and tunneling resistance on the conductivity of carbon nanotubes nanocomposites. *Polym. Compos.* **41**(2), 748–756 (2020).

[13] Zare, Y. & Rhee, K. Y. A simple methodology to predict the tunneling conductivity of polymer/CNT nanocomposites by the roles of tunneling distance, interphase and CNT waviness. *RSC Adv.* **7**(55), 34912–34921 (2017).

[14] Kim, H., Abdala, A. A. & Macosko, C. W. Graphene/polymer nanocomposites. *Macromolecules* **43**(16), 6515–6530 (2010).

[15] Begum, S. *et al.* Investigation of morphology, crystallinity, thermal stability, piezoelectricity and conductivity of PVDF nanocomposites reinforced with epoxy functionalized MWCNTs. *Compos. Sci. Technol.* **211**, 108841 (2021).

[16] Chanda, A., Sinha, S. K. & Datla, N. V. Electrical conductivity of random and aligned nanocomposites: Theoretical models and experimental validation. *Compos. Part A Appl. Sci. Manuf.* **149**, 106543 (2021).

[17] Colonna, S. *et al.* Effect of morphology and defectiveness of graphene-related materials on the electrical and thermal conductivity of their polymer nanocomposites. *Polymer* **102**, 292–300 (2016).

[18] Zare, Y. & Rhee, K. Y. A simple model for electrical conductivity of polymer carbon nanotubes nanocomposites assuming the filler properties, interphase dimension, network level, interfacial tension and tunneling distance. *Compos. Sci. Technol.* **155**, 252–260 (2018).

[19] Zare, Y., Rhee, K. Y. & Park, S. J. Simulation of tunneling distance and electrical conductivity for polymer carbon nanotubes nanocomposites by interphase thickness and network density. *Polym. Compos.* **41**(6), 2401–2410 (2020).

[20] Zare, Y. & Rhee, K. Y. Expression of characteristic tunneling distance to control the electrical conductivity of carbon nanotubes-reinforced nanocomposites. *J. Mater. Res. Technol.* **9**(6), 15996–16005 (2020).

[21] Clingerman, M. L., King, J. A., Schulz, K. H. & Meyers, J. D. Evaluation of electrical conductivity models for conductive polymer composites. *J. Appl. Polym. Sci.* **83**(6), 1341–1356 (2002).

[22] Chang, L., Friedrich, K., Ye, L. & Toro, P. Evaluation and visualization of the percolating networks in multi-wall carbon nanotube/epoxy composites. *J. Mater. Sci.* **44**(15), 4003–4012 (2009).

[23] Kara, S., Arda, E., Dolastir, F. & Pekcan, Ö. Electrical and optical percolations of polystyrene latex–multiwalled carbon nanotube composites. *J. Colloid Interface Sci.* **344**(2), 395–401 (2010). 20106484 10.1016/j.jcis.2009.12.056

[24] Zare, Y. Modeling the strength and thickness of the interphase in polymer nanocomposite reinforced with spherical nanoparticles by a coupling methodology. *J. Colloid Interface Sci.* **465**, 342–346 (2016). 26704592 10.1016/j.jcis.2015.09.025

[25] Zare, Y., Rhee, K. Y. & Park, S.-J. Predictions of micromechanics models for interfacial/interphase parameters in polymer/metal nanocomposites. *Int. J. Adhes. Adhes.* **79**, 111–116 (2017).

[26] Zare, Y. & Rhee, K. Y. Prediction of tensile modulus in polymer nanocomposites containing carbon nanotubes (CNT) above percolation threshold by modification of conventional model. *Curr. Appl. Phys.* **17**(6), 873–879 (2017).

[27] Baek, K., Shin, H. & Cho, M. Multiscale modeling of mechanical behaviors of nano-SiC/epoxy nanocomposites with modified interphase model: Effect of nanoparticle clustering. *Compos. Sci. Technol.* **203**, 108572 (2021).

[28] Gao, C., Zhan, B. & Yang, C. A static/dynamic micromechanical model of graphene-reinforced polymer matrix nanocomposites with consideration of the nanoscale interphase. *Mech. Mater.* **157**, 103838 (2021).

[29] Hassanzadeh-Aghdam, M., Mahmoodi, M., Ansari, R. & Darvizeh, A. Interphase influences on the mechanical behavior of carbon nanotube–shape memory polymer nanocomposites: A micromechanical approach. *J. Intell. Mater. Syst. Struct.* **30**(3), 463–478 (2019).

[30] Zare, Y. & Rhee, K. Y. The strengthening efficacy of filler/interphase network in polymer halloysite nanotubes system after mechanical percolation. *J. Mater. Res. Technol.* **15**, 5343–5352 (2021).

[31] Zare, Y. Effects of imperfect interfacial adhesion between polymer and nanoparticles on the tensile modulus of clay/polymer nanocomposites. *Appl. Clay Sci.* **129**, 65–70 (2016).

[32] Zare, Y. & Rhee, K. Y. The mechanical behavior of CNT reinforced nanocomposites assuming imperfect interfacial bonding between matrix and nanoparticles and percolation of interphase regions. *Compos. Sci. Technol.* **144**, 18–25 (2017).

[33] Zare, Y. & Rhee, K. The roles of polymer-graphene interface and contact resistance among nanosheets in the effective conductivity of nanocomposites. *Appl. Math. Mech.* **44**(11), 1941–1956 (2023).

[34] Shin, H., Yang, S., Choi, J., Chang, S. & Cho, M. Effect of interphase percolation on mechanical behavior of nanoparticle-reinforced polymer nanocomposite with filler agglomeration: A multiscale approach. *Chem. Phys. Lett.* **635**, 80–85 (2015).

[35] Balberg, I., Azulay, D., Toker, D. & Millo, O. Percolation and tunneling in composite materials. *Int. J. Mod. Phys. B* **18**(15), 2091–2121 (2004).

[36] Qiao, R. & Brinson, L. C. Simulation of interphase percolation and gradients in polymer nanocomposites. *Compos. Sci. Technol.* **69**(3), 491–499 (2009). 10.1016/j.compscitech.2009.04.005 PMC2702785 20161273

[37] Li, J. & Kim, J.-K. Percolation threshold of conducting polymer composites containing 3D randomly distributed graphite nanoplatelets. *Compos. Sci. Technol.* **67**(10), 2114–2120 (2007).

[38] Zare, Y., Rhee, K. Y. & Hui, D. Modeling of electrical conductivity for graphene-based systems by filler morphology and tunneling length. *Diam. Relat. Mater.* **134**, 109782 (2023).

[39] Vatani, M., Zare, Y., Gharib, N., Rhee, K. Y. & Park, S.-J. Simulating of effective conductivity for grapheme–polymer nanocomposites. *Sci. Rep.* **13**(1), 5907 (2023). 37041268 10.1038/s41598-023-32991-w PMC10090123

[40] Zare, Y. & Rhee, K. Y. Electrical conductivity of graphene-containing composites by the conduction and volume share of networked interphase and the properties of tunnels applicable in breast cancer sensors. *J. Mater. Sci.* **57**(37), 17637–17648 (2022).

[41] Yanovsky, Y. G., Kozlov, G. & Karnet, Y. N. Fractal description of significant nano-effects in polymer composites with nanosized fillers. Aggregation, phase interaction, and reinforcement. *Phys. Mesomech.* **16**(1), 9–22 (2013).

[42] Deng, F. & Zheng, Q.-S. An analytical model of effective electrical conductivity of carbon nanotube composites. *Appl. Phys. Lett.* **92**(7), 071902 (2008).

[43] Weber, M. & Kamal, M. R. Estimation of the volume resistivity of electrically conductive composites. *Polym. Compos.* **18**(6), 711–725 (1997).

[44] Li, J. *et al.* Correlations between percolation threshold, dispersion state, and aspect ratio of carbon nanotubes. *Adv. Funct. Mater.* **17**(16), 3207–3215 (2007).

[45] Zare, Y., Rhee, K. Y. & Hui, D. Predicting of electrical conductivity for graphene-filled products by tunneling mechanism and interphase piece to enhance the performance of breast cancer biosensors. *Eur. Phys. J. Plus* **137**(8), 980 (2022).

[46] Stankovich, S. *et al.* Graphene-based composite materials. *Nature* **442**(7100), 282–286 (2006). 16855586 10.1038/nature04969

[47] Gao, C. *et al.* Graphene networks with low percolation threshold in ABS nanocomposites: Selective localization and electrical and rheological properties. *ACS Appl. Mater. Interfaces* **6**(15), 12252–12260 (2014). 24969179 10.1021/am501843s

[48] Tu, Z. *et al.* A facile approach for preparation of polystyrene/graphene nanocomposites with ultra-low percolation threshold through an electrostatic assembly process. *Compos. Sci. Technol.* **134**, 49–56 (2016).

[49] Goumri, M., Lucas, B., Ratier, B. & Baitoul, M. Electrical and optical properties of reduced graphene oxide and multi-walled carbon nanotubes based nanocomposites: A comparative study. *Opt. Mater.* **60**, 105–113 (2016).

[50] Zhang, H.-B. *et al.* Electrically conductive polyethylene terephthalate/graphene nanocomposites prepared by melt compounding. *Polymer* **51**(5), 1191–1196 (2010).

[51] Feng, C. & Jiang, L. Micromechanics modeling of the electrical conductivity of carbon nanotube (CNT)–polymer nanocomposites. *Compos. Part A Appl. Sci. Manuf.* **47**, 143–149 (2013).

[52] Messina, E. *et al.* Double-wall nanotubes and graphene nanoplatelets for hybrid conductive adhesives with enhanced thermal and electrical conductivity. *ACS Appl. Mater. Interfaces* **8**(35), 23244–23259 (2016). 27538099 10.1021/acsami.6b06145

